# Uncommon Progressive Systemic Tetanus: A Case Report

**DOI:** 10.7759/cureus.38383

**Published:** 2023-05-01

**Authors:** Reinaldo Sanchez-Grillo, Esteban Zavaleta-Monestel, Eduardo Ruiz-Munguia, José Pablo Díaz-Madriz, Carolina Rojas-Chinchilla

**Affiliations:** 1 Critical Care Medicine, Hospital Clínica Bíblica, San José, CRI; 2 Department of Pharmacy, Hospital Clínica Bíblica, San José, CRI; 3 Internal Medicine, Hospital Clínica Bíblica, San José, CRI; 4 Faculty of Pharmacy, Universidad de Costa Rica, San José, CRI

**Keywords:** dtap vaccine, spasm, opisthotonus, lacosamide, benzodiazepines, tetanus

## Abstract

Tetanus is a bacterial infection caused by the toxin of *Clostridium tetani*. While it primarily affects newborns, people with incomplete vaccination schedules, it can also impact people of any age, especially in developing countries. Even though in the last 20 years several initiatives have been implemented worldwide to reduce the impact of this disease, regions like South Asia and sub-Saharan Africa have registered mortality rates highest since 2015-2019. In Latin America, regional immunization coverage rates were reported at 89% in 2017 for diphtheria-tetanus toxoid and pertussis (DTP-3), although Costa Rica has reported decreased coverage rates of the national immunization schedule from 2019 to 2021.

In this case study, we present a 53-year-old woman from Puntarenas, Costa Rica diagnosed with progressive systemic tetanus who developed status epilepticus. She previously was assessed in a central hospital of Costa Rica for paresthesia in her right upper limb of three months of duration, myoclonus and difficulty walking in the last weeks; the presumed diagnosis was Guillain-Barré syndrome. During her hospitalization she had three generalized tonic-clonic seizures treated with diazepam and phenytoin.

Since there was no improvement, she was transferred to our medium-sized private hospital for the treatment of painful spasms and weakness in the lower limbs. On initial evaluation, no injury was found. She was initially treated with midazolam and magnesium sulfate for presenting seizures-like spasms in the lower limbs and then generalized without loss of consciousness for up to 15 minutes, mainly associated with desaturation, tachycardia and tachypnea. In the differential diagnosis, muscle contractions linked to hypocalcemia, neurosyphilis and epilepsy were ruled out. Despite this, magnetic resonance imaging showed fractures in T11, L1 and L2. Mainly due to the presence of spasms, opisthotonos and history of seizures and a wound on the hand four months ago, she was diagnosed with tetanus.

Among the initial management, tetanus toxoid (Td), antimicrobial therapy, and human antitetanic immunoglobulin (HTIG) were administered, which partially improved the patient’s condition, although she remained dependent on the infusions. On the sixth week of hospitalization, the patient developed status epilepticus which is explained by the magnetic resonance findings that show subacute bi-occipital infarcts caused by hypoxia from the previous crises. Lacosamide therapy reversed the condition and kept the patient free of seizures.

It was necessary to carry out a lumbar osteosynthesis which was highly favorable to stabilize the patient's condition. The frequency and intensity of the spasms were gradually reduced, which allowed the gradual suspension of the infusions and the benzodiazepine overlap intravenous (IV) to oral (PO). The patient now has only self-limiting spasms and her maintenance therapy consists of lacosamide and oral clonazepam.

This case highlights the importance of considering tetanus in the differential diagnosis even if the vaccination schedule is complete, especially if there are spasms, convulsions, or a history of wounds or bites. It is important to monitor this type of report to reconsider and update the key elements in the prevention, diagnosis, management, and treatment of tetanus; as well as improve access to essential medicines, including the HTIG, and the patient's prognosis in terms of symptom resolution and associated sequelae.

## Introduction

The tetanus toxin which is produced by *Clostridium tetani* (*C. tetani*) can cause an acute infection that is often characterized by rigidity and convulsive spasms of skeletal muscles [1]. Tetanus neurotoxin (TeNT) initially binds to presynaptic terminals at the neuromuscular junction, is transported to the spinal cord, and is transferred to inhibitory presynaptic terminals surrounding these motor neurons. TeNT then destroys a vesicular synaptic membrane protein (VAMP), inactivating inhibitory neurotransmission. This process increases the excitability and activation of the motoneurons in question. Systemic intoxication results in clinically generalized muscle contractions similar to tetanus [2].

Tetanus is classified into four categories: neonatal, local, cephalic, and generalized, with the latter being the most severe and having the highest risk of complications. The most frequent form of the disease is generalized tetanus (>80% of cases) which causes pain, headache, stiffness, rigidity, opisthotonos, and painful spasms, which can lead to laryngeal obstruction, even some slight stimuli such as noise, touch, and minor medical procedures could trigger those symptoms. TeNT's effects are not limited to the motor system, since autonomic dysfunction is common in generalized tetanus, with episodes of tachycardia, hypertension, and sweating, sometimes rapidly alternating with bradycardia and hypotension [3]. Also, spasms are most noticeable during the first two weeks of the disease; autonomic disturbance usually begins a few days after spasms and peaks during the second week, and rigidity can last longer than both spasms and autonomic upset [4].

The disease is highly preventable, primarily through immunization and proper wound care, in fact, mass vaccination has reduced incidence and mortality by 89% since 1990 worldwide [3,5,6]. However, in 2017 globally 38,000 people died of tetanus and 49% were under five years of age. Currently, the majority of new cases arise in countries of South Asia and sub-Saharan Africa, which represent 82% of the cases reported worldwide. By 2017, Somalia and South Sudan had tetanus case rates greater than 10 per 100,000 people and only 42% and 49% of the population under one year of age received the third dose of the vaccine [6]. It remains an important public health problem in many parts of the world, especially in countries where immunization coverage is low, and uncertain labor practices are common [5]. In this case report, we present a patient who was diagnosed with severe tetanus and developed status epilepticus in a private hospital in Costa Rica.

## Case presentation

We present a case of a 53-year-old female with past medical history of hypertension, controlled type 2 diabetes mellitus, immunocompetent, with the complete vaccination schedule including the diphtheria, tetanus and pertussis vaccine (DTaP) schedule. Among her personal surgical history, she only had a cesarean section in 2004. Despite having no personal or family history of psychiatric illnesses, she referred emotional and physical overload as a caregiver of two relatives who are in the terminal phase of cancer.

She was assessed in a rural hospital for paresthesia in her right upper limb of three months of duration and onset of myoclonus and difficulty walking during the prior three weeks. There she was admitted for a presumed diagnosis of Guillain-Barré syndrome. During her hospitalization, she reportedly had three generalized tonic-clonic seizures treated with diazepam and phenytoin. Since there was no improvement, she was transferred to a more advanced care medium-sized hospital in the city for the treatment of painful contractions and weakness in the lower limbs.

During the first day of hospitalization, the physical examination was performed and no lesions or injuries were found, also, in the initial interview the patient did not mention any type of pre-existing injury. As well, she had multiple seizures-like spasms in lower limbs and then generalized to upper limbs for up to 15 minutes without loss of consciousness, with perioral cyanosis with oxygen saturation (SpO_2_) of 68%, non-reactive mydriatic pupils, tachycardiac, tachypneic, diaphoretic, normoglycemic and normotensive.

**Figure 1:**
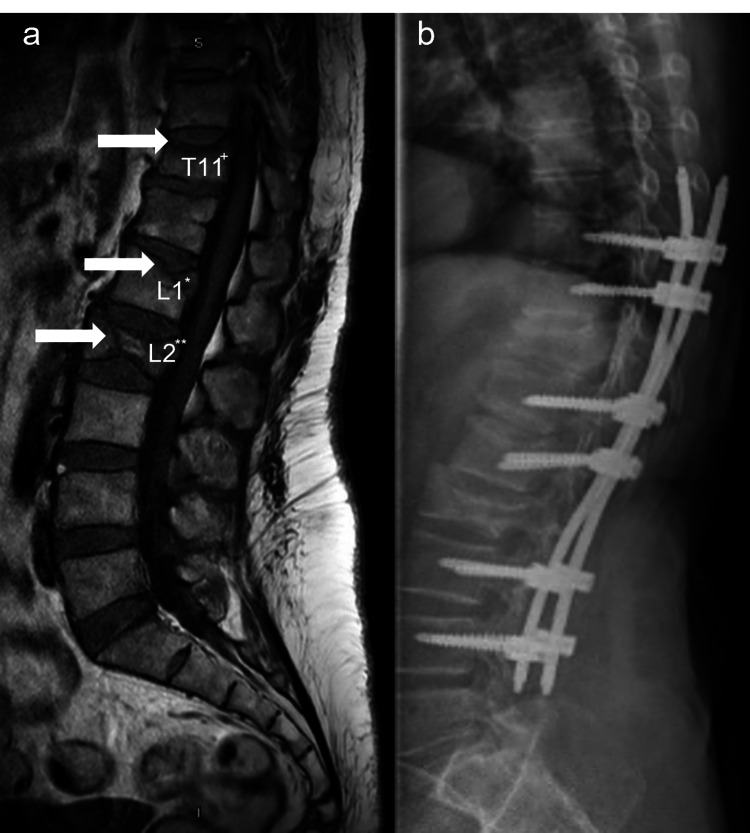
Magnetic resonance imaging of spinal fractures caused by generalized tetanus. a) Spinal fractures caused by progressive tetanus. b) Lumbar osteosynthesis performed at T11, L1 and L2. +: mild fracture of T11. *: annular fracture of the L1 plateau. **: burst fractures in L2.

The spasms were treated with doses of 5-15mg IV midazolam; the patient never showed signs of sedation or drowsiness caused by this drug. Also, the existence of a postictal period was ruled out. It was noticed that some of the spasms were triggered by sounds and extreme stimuli and preceded by low back pain or subsequent movement. The magnetic resonance imaging (MRI) showed burst fractures at L2, an annular fracture of the L1 plateau and a slight fracture of T11, associated with the spasms since the patient had no history of trauma (Figure 1).

On the fourth day, the labs suggested an infectious disease with leukocytosis (18.5 x10^3 ^cells/μL), neutropenia (11.5 x10^3 ^cells/μL), increased levels of C reactive protein (CRP) at 30.6mg/L and procalcitonin (PCT) at 0.231ng/mL, hypokalemia (2.61mmol/L), hypomagnesemia (1.59mg/dL), and hypocalcemia (8.37mg/dL). In the differential diagnosis, muscle contractions linked to hypocalcemia were ruled out due to negative Chvostek and Trousseau signs, studies were also performed for neurosyphilis, however total anti-treponema pallidum antibodies were non-reactive. Finally, the MRI showed no signs of epilepsy.

In addition, the patient was interviewed again to inquire about the possible source of infection, she indicated having had a wound on the hand four months ago, but when examined there were no signs of this injury. Mainly due to the presence of spasms in the lower limbs and lumbar muscles, opisthotonos, the clinical history of generalized seizures and history of the wound mentioned by the patient, she was diagnosed with severe tetanus [7].

**Figure 2:**
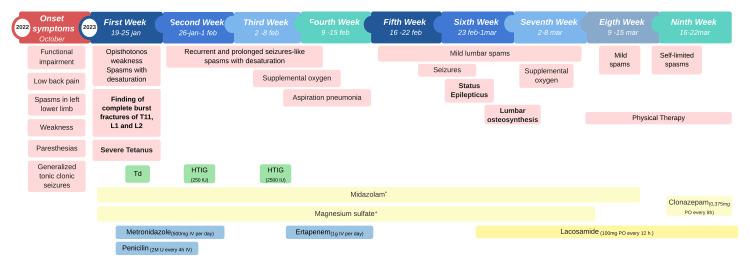
Timeline of severe tetanus progression, sequelae, and treatment Clinical manifestations are represented in red, antibiotic therapy in blue, immunoglobulin and vaccines in green, and treatment of spasms and status epilepticus in yellow. *The midazolam infusions used were high doses 90-100mg IV in 100mL of saline at 6-12mL/h, maintenance dose 45mg IV in 100mL of saline at 6ml/h or low doses 15mg IV in 100mL of saline at 3mL/h depending on the evolution of the patient. +The magnesium sulfate infusions used were high doses 12g IV in 250mL of dextrose 5% at 7mL/h, maintenance dose 12g IV in 250mL of dextrose 5% at 20mL/h or low doses 4g IV in 250mL of dextrose 5% at 4mL/h depending on the evolution of the patient. HTIG: human tetanus immunoglobulin, Td: tetanus toxoid

Due to her seizures-like spasms, there was a fear of fatal respiratory failure, leading to her reassessment and admission to the intensive care unit (ICU). The tetanic toxoid (Td) was given immediately (the patient refers she has a complete DTaP schedule, and the last booster was given six years ago). Midazolam and magnesium sulfate were continued as well, as the initiation of antimicrobial therapy with metronidazole 500mg IV every 24 hours and penicillin 2 million U IV every four hours for seven days (Figure 2). The levels of CRP and PCT decreased, and the leukocytes were no longer altered. Also, the frequency and severity of spasms significantly decreased, although not entirely resolved.

One dose of human tetanus immunoglobulin (HTIG) 250 IU was given on the eighth day due to the limited availability countrywide. On the night of the 14th day, the patient suffered generalized crises with episodes of oxygen desaturation. It was possible to get more HTIG, therefore the following day, an additional dose of 2500 IU of HTIG was given. After high-dose HTIG, the patient gradually improved with reductions in the lower back and back pain; therefore, she was monitored in intermediate care. The patient did not present generalized spasms during the following three days, however, after reducing the infusions of benzodiazepines and magnesium sulfate, she suffered multiple crises with SpO2 in 68%, which forced the restart of therapy. On day 21, the patient experienced a crisis in which she aspirated developing bronchopneumonia treated with ertapenem 1g IV every 24 hours for a total of six days, other complications occurred such as persistent spasms in the lower limbs and lower back, urinary tract infection associated with Foley catheter, and even orthopedic evaluation to consider alternative treatments.

On the sixth week, the patient presented several generalized tonic-clonic seizures lasting from 45 seconds to two minutes treated with midazolam 10mg IV, propofol 50-60mg IV, and magnesium sulfate 4g in 250mL of dextrose 5% IV for 20 minutes. Due to the recurrent seizures, it was determined that the patient was in status epilepticus and was monitored in the ICU. The electroencephalogram recorded bi-anterior spikes and generalized bursts of sharp waves. A brain MRI showed subacute bi-occipital infarcts secondary to periods of hypoxia related to her previous crises. Therefore, it was necessary to increase the maintenance dose of midazolam to 90-100mg IV in 100mL of saline at 6-12mL/h and magnesium sulfate to 12g IV in 250mL of dextrose 5% at 7mL/h, lacosamide 100mg every 12 hours orally was maintained to control seizures.

Also, an osteosynthesis of the lumbar spine was performed for T1, L1, and L2 burst fractures to improve the patient's condition (Figure 1). In the following days, the patient started physical rehabilitation therapy and midazolam infusions were decreased to 15mg IV in 100mL of saline at 3mL/h and magnesium sulfate 4g in 250mL of dextrose 5% at 4mL/h until discontinued. By the ninth week the patient improved the strength of the lower and upper limbs, fed herself and was able to stand up with help, with very mild sporadic crises due to a dysautonomic component but mainly emotional, for which she is maintained with clonazepam drops 2.5mg/mL three drops PO every eight hours, finally with hospital discharge.

## Discussion

Tetanus is a potentially life-threatening infection related to a neurotoxin produced by anaerobic bacillus *C. tetani*, where most reported cases are associated with newborns and mothers who do not have adequate access to immunizations and others who do not have the complete vaccination schedule, especially in developing countries [5,8].

Even though in the last 20 years, several initiatives have been implemented worldwide to reduce the impact of this disease, there are still some regions more affected than others [9,10]. For example, South Asia and sub-Saharan Africa have registered mortality rates highest since 2015, with no improvement yet in 2019 [6]. In Latin America, regional immunization coverage rates were reported at 89% in 2017 for diphtheria-tetanus toxoid and pertussis (DTP-3), however, different scenarios exist between some countries, which may also be related to the new rate of tetanus infections [6,11].

Particularly, from 1998 to 2011, only one case of tetanus was reported per 100,000 people per year in Costa Rica. Subsequently, the average dropped to 0 cases per 100,000 people per year from 2015 to 2019. It should be mentioned that there are no data on the number of deaths from tetanus in the country and the statistical bias of unreported cases should be considered [6]. Recently, the country's health authorities have reported decreased coverage rates of the national immunization schedule from 2019 to 2021 [12].

In Costa Rica, the complete immunization schedule in children under 10 years of age consists of administering the dose of the basic schedule of pentavalent vaccine (DTaP/Hib/IPV) at two, four, and six months of age. Then, booster doses at 15 months with DTaP/Hib/IPV, at four years with DTaP and at 10 years with Td [13]. In the case of adults with the complete children's scheme, the Td reinforcement is placed every 10 years. If the adult has an incomplete scheme, the Td 0-1-6 scheme is applied, with 0 being the first dose applied, one month and six months after the first application, the booster dose is applied every 10 years. This scheme was updated in 2022, previously only DTaP and Td were available [14].

Otherwise, the diagnosis of tetanus is primarily clinical and frequently simple. However, it can also be difficult, particularly in countries where this infection is currently extremely uncommon as is the case in Costa Rica, a late diagnosis may lead to therapy delays, resulting in a worse outcome. While a positive culture of *C. tetani* from a wound sample can help confirm the diagnosis, it is not always essential, as in this particular case where no culture was available [1,15]. Meningitis, drug-induced dystonias, trismus due to dental infections, seizure, hypocalcemia, rabies, strychnine poisoning, stroke, malignant neuroleptic syndrome, and stiff person syndrome are some of the differentials for tetanus [16].

The target of tetanus therapeutic management is to prevent disease progression by using therapies that inhibit toxin production and neutralize the free toxin. Supportive care and symptomatic treatment are provided to control spasms and autonomic instability, protect airways, provide adequate ventilation, reduce the triggering stimuli, and address metabolic issues [17,18].

Once a diagnosis has been made, the recommended first step is to neutralize circulating tetanus toxins by administration of HTIG and, in the event of a shortage, an equine tetanus immunoglobulin could be used. There is no consensus on an ideal dose, as 500 IU of HTIG has been reported to be as effective as 3000-6000 IU IM [17,19]. In this case, due to a local shortage of HTIG, 250 IU was administered first and an additional 2500 IU was administered 11 days after diagnosis. Although equine immunoglobulin is an option in shortage settings, this medical center was only able to obtain HTIG [17]. The frequency of spasms was reduced, absence of generalized crises for a few days. The Td vaccine is also recommended as part of initial therapy since tetanus does not confer protective immunity [19].

Antimicrobial therapy is also a key element in preventing the progression of this infection. The antibiotics with the most evidence in the management of tetanus are metronidazole 500mg IV every six hours for seven to 10 days or penicillin 20 million units per day IV in divided doses every four to six hours for seven to 10 days [19,20]. One study showed that comparing treatment with benzathine benzylpenicillin (1.5 million U every eight hours IM for seven to 10 days) or metronidazole (500mg every six hours PO) suggested that metronidazole therapy is optimal, as it is associated with lower mortality rates in patients with tetanus [21]. Taking into account the severity of the patient's clinical picture, both antibiotics were indicated as part of the therapy.

Standard therapy for control of spasms is magnesium sulfate at a maximum loading dose of 5g and a continuous infusion of 2 to 3g/hour until the spasm is controlled, also benzodiazepines such as diazepam or midazolam, given an intravenous infusion of 5 to 15mg/hour. These drugs helped control autonomic dysthymia and reduce seizures [18,20].

After HTIG administration, infusions of magnesium sulfate and midazolam were considerably reduced, however, seizures-like spasms continued, so infusions were maintained at high or maintenance doses. Lumbar osteosynthesis was a key point in the suspension of the infusions since it decreased the intensity and frequency of the spasms afterward, allowed starting physiotherapy, and improved muscle strength. Overlapping midazolam to oral clonazepam was performed to control sporadic self-limited spasms.

Regarding the preservation of the airway, patients usually require invasive ventilation methods such as mechanical ventilation, tracheostomy, or others due to the severity of the crises they experience associated with desaturation [17]. The patient did not present major ventilatory complications, only transient desaturations in her crises managed with supplemental oxygen and aspiration pneumonia treated with ertapenem. Still, she did not require invasive ventilation as in most cases of severe tetanus.

Thus, cases of severe tetanus that trigger status epilepticus have been reported. This is a medical emergency that, if refractory, increases the morbidity and mortality rate. Linked to this, therapeutic management is complex if it is not resolved with the first or second lines of treatment. In 2014, lacosamide was approved as monotherapy for focal epilepsy. This drug has rapid absorption, 100% bioavailability, is equivalent to an intravenous formulation, has few drug interactions, and has better seizure control. In this case, from the beginning of treatment with lacosamide, the refractory state was completely controlled so it will be maintained as maintenance therapy [22].

Typically, the duration of tetanus is around four to six weeks, but it may extend beyond that time; this patient remains hospitalized and has been in recovery for 62 days, and more than four months have passed since the onset of symptoms. It has been reported that muscular spasms ceased up to six months or even a year after discharge, with a complete absence of any other tetanus symptoms [17].

Undoubtedly, compliance with vaccination schedules, as well as the application of boosters every 10 years, are essential at the public health level in the prevention and recurrence of diseases such as tetanus, however, cases have been reported in which these preventive measures are followed and even, so patients acquire this infection. This type of therapeutic failure due to the vaccine or the low levels of tetanus antitoxin generated could be associated with factors external to the vaccine such as immune or nutritional status or alterations in cold chains [16].

## Conclusions

Given the significant efforts of Latin American countries such as Costa Rica in strengthening vaccination schemes and compliance with boosters, the appearance of potentially fatal and preventable infections such as tetanus in vaccinated populations is worrisome. As in this case, other patients with complete vaccination schedules have been reported who develop tetanus, which creates a complex scenario for a correct differential diagnosis. In general, attention should be paid to this type of case to reestablish the key elements in the prevention, diagnosis, management, and treatment of tetanus, as well as to improve access to essential drugs such as HTIG to achieve a better prognosis for the patient concerning the resolution of symptoms and associated sequelae.
